# Lysosomotropic challenge of mast cells causes intra-granular reactive oxygen species production

**DOI:** 10.1038/s41420-019-0177-3

**Published:** 2019-05-15

**Authors:** Aida Paivandy, Jens Eriksson, Fabio Rabelo Melo, Mikael E. Sellin, Gunnar Pejler

**Affiliations:** 1 Uppsala University, Department of Medical Biochemistry and Microbiology, Uppsala, Sweden; 2 Science for Life Laboratory, Uppsala University, Department of Medical Biochemistry and Microbiology, Uppsala, Sweden; 3 Swedish University of Agricultural Sciences, Department of Anatomy, Physiology and Biochemistry, Uppsala, Sweden

## Abstract

Mast cells contribute to the pathology of allergic and other disorders. Strategies to interfere with harmful mast cell-related activities are therefore warranted. Previously we established a principle for inducing selective apoptosis of mast cells, by the use of lysosomotropic agents that cause secretory granule permeabilization, leading to production of reactive oxygen species (ROS). However, the mechanism of ROS production has not been known. Here we addressed this issue. Live microscopy analysis showed that the secretory granules comprise major subcellular compartments for ROS production in response to mefloquine. As further signs for the primary involvement of secretory granules, both ROS production and cell death was blunted in mast cells lacking serglycin, a secretory granule-restricted proteoglycan. Inhibition of granule acidification caused an essentially complete blockade of granule permeabilization, ROS production and cell death in response to mefloquine. ROS production was also attenuated in the presence of an iron chelator, and after inhibition of either granzyme B or the ERK1/2 MAP kinase signaling pathway. Together, our findings reveal that the mast cell secretory granules constitute major sites for ROS production in mast cells subjected to lysosomotropic challenge. Moreover, this study reveals a central role for granule acidification in ROS generation and the pro-apoptotic response triggered downstream of secretory granule permeabilization.

## Introduction

Mast cells are long-lived tissue resident immune cells that originate from hematopoietic precursors in the bone marrow. They circulate in the blood as immature progenitors and, upon transmigration into peripheral tissues, they mature under the influence of local growth factors^1^. Fully mature mast cells are filled with lysosome-like organelles known as secretory granules that are rich in preformed bioactive compounds, including biogenic amines, serglycin proteoglycans, cytokines and various lysosomal and mast cell-specific proteases^2^. Owing to their abundant presence in strategic locations at host–environment interfaces, mast cells can serve as immune sentinel cells to respond to invaders^3^. However, mast cells are also infamous for their detrimental roles in orchestrating the inflammatory responses in numerous pathological conditions including allergic disorders (e.g., atopic dermatitis and allergic rhinitis)^4^, chronic inflammatory diseases (e.g., asthma and arthritis)^5–7^ and different types of cancers^8–10^.

Given the multifaceted and central role of mast cells in the pathogenesis of various inflammatory diseases and malignancies, the targeting of mast cells has emerged as an attractive and broadly applicable therapeutic strategy^11,12^. In this respect, we have previously introduced a novel approach to induce mast cell apoptosis through the use of lysosomotropic agents, which have been shown to cause secretory granule permeabilization^13–15^. Moreover, we have demonstrated that cell death in response to lysosomotropic agents, including mefloquine (an anti-malaria drug), shows selectivity for mast cells and is associated with reactive oxygen species (ROS) generation^13,16^. However, the mechanism behind ROS generation in mast cells in response to granule permeabilization has not been revealed.

In the present study we aimed to determine the mechanism underlying ROS production in response to granule permeabilization of mast cells. By live imaging analysis we show that ROS production in response to lysosomotropic agents predominantly occurs within the secretory granule compartment of mast cells. In further support for intra-granular production of ROS, both ROS production and cell death was blunted in mast cells lacking serglycin, a secretory granule-restricted proteoglycan. Furthermore, our findings indicate that granule acidification has a central role in the responses to lysosomotropic challenge. Finally, we show that iron is a source of ROS in mast cells and that granzyme B has a role in the pathway driving ROS production.

## Results

### ROS production in response to lysosomotropic challenge occurs within mast cell secretory granules

We demonstrated previously that the lysosomotropic agent, mefloquine, has a strong and selective pro-apoptotic effect on murine and human mast cells, and that cell death in response to mefloquine is associated with the production of ROS^13^. However, the mechanism of ROS production in response to lysosomotropic challenge has not been known. In our previous studies we found that the ROS production was non-sensitive to alpha-tocopherol^13^, a compound that blocks mitochondrial ROS production. This led us to hypothesize here that mitochondria are not the major subcellular compartments responsible for ROS production. Since mast cell secretory granules are known to be targets for lysosomotropic agents^13,15,17^ we instead hypothesized that the ROS production in response to lysosomotropic challenge could occur within the secretory granules. To evaluate this hypothesis, we used live confocal imaging.

**Fig. 1:**
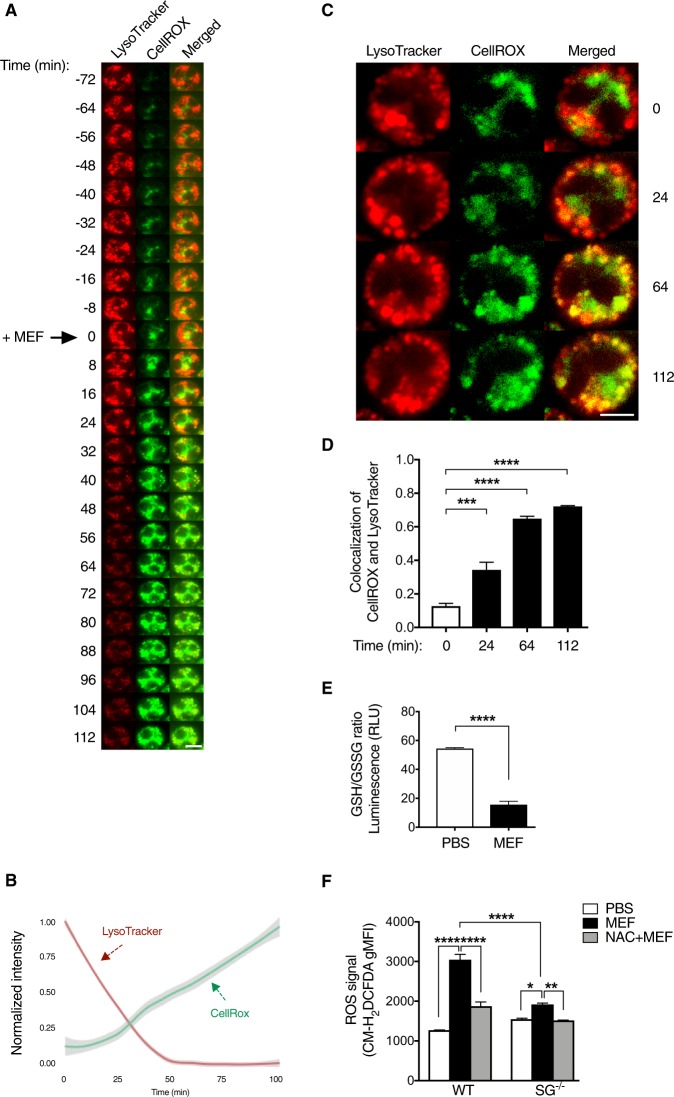
Intracellular ROS induced by mefloquine are localized inside the mast cell granules. **a** Representative live confocal images illustrating the granule damage and production of ROS in mefloquine-treated mast cells. BAM-anchored bone marrow-derived mast cells (BMMCs) were pre-incubated with LysoTracker Red DND-99 (to label granules) and CellROX Deep Red (green; to monitor ROS production) for 30 min at 37 °C. Cells were then washed and live confocal imaging was performed immediately. After an initial image recording for 72 min (t = −72), mefloquine (20 μM) was added to the cells (t 0) and image recording continued for 112 min (t = 112). Scale bar = 5 µm. **b** Kinetics of LysoTracker and CellROX signal intensities in mefloquine-treated BMMCs, representing the intensity from 800–1000 cells in total. **c** Representative live confocal images illustrating intracellular localization of ROS (CellROX signal in green) within mast cell secretory granules (LysoTracker signal in red) upon mefloquine treatment. Intensities at each time point have been normalized to accentuate the localization of signal. Scale bar = 5 µm. **d** Quantification of the colocalization of CellROX with LysoTracker. Colocalization analysis was carried out on single confocal slices from four independent full fields of view at the indicated time points, representing 800–1000 cells in total. Bars indicate mean Mander’s M1 coefficient ± SEM (*n* = 4), one-way ANOVA with Dunnett’s multiple comparisons test. **e** GSH/GSSG ratio in response to mefloquine treatment. BMMCs were incubated ± mefloquine (20 µM) for 30 min. Cell lysates were prepared and incubated with Luciferin Generation Reagent (30 min) followed by incubation with Luciferin Detection Reagent (15 min). Subsequently, luminescence was measured using a TECAN microplate reader and the GSH/GSSG ratio was determined. Mean ± SEM (*n* = 3), unpaired, two-tailed Student’s *t*-test. **f** ROS levels in wild type (WT) and serglycin-deficient (SG^−/−^) mast cells. BMMCs were preincubated ± 8 mM N-acetylcysteine (NAC) for 2 h followed by incubation ± 20 μM mefloquine (MEF). After 30 min, cells were washed and incubated (30 min) with CM-H_2_DCFDA. Cells were then washed and the fluorescence intensity was immediately assessed by flow cytometry. The bars represent means ± SEM of the geometric mean fluorescence intensity (gMFI) for CM-H_2_DCFDA (*n* = 4)

As shown in Fig. 1a, prior to mefloquine treatment, mast cell granules were clearly visible (labeled with LysoTracker) and intact, and only low levels of intracellular ROS (monitored with CellROX) were detected. Upon addition of mefloquine, a rapid elevation of ROS production was seen. By contrast, the LysoTracker signal showed a marked reduction at the same time (Fig. 1a, b; Suppl. Video 1), indicating damage to the mast cell granules. To determine if the ROS generated in mefloquine-treated mast cells arose from mast cell granules, we monitored the colocalization of LysoTracker and CellROX. At the time of mefloquine addition (t 0), a low extent of LysoTracker/CellROX colocalization was seen (Fig. 1c, d). However, as time passed (t 24, 64 and 112 min), a significantly higher extent of LysoTracker/CellROX colocalization was observed (Fig. 1c, d and Suppl. Video 1). These findings suggest that the mast cell granules constitute major subcellular sites for ROS production in response to mefloquine.

Glutathione (GSH) is an important ROS scavenger during oxidative stress. Given that GSH depletion occurs as an early hallmark event of cell death in response to various apoptotic stimuli^18,19^, the GSH/GSSG (GSSG represents oxidized glutathione) ratio was assessed following mefloquine treatment of mast cells. As shown in Fig. 1e, incubation of mast cells with mefloquine caused a decrease in the GSH/GSSG ratio, verifying that lysosomotropic challenge of mast cells causes oxidative stress.

As another approach to assess the importance of the secretory granules in ROS production upon lysosomotropic challenge, we compared the ROS production in wild-type (WT) and serglycin-deficient (serglycin^−/−^) mast cells. Serglycin is a proteoglycan exclusively located in the secretory granules of mast cells^20^, and effects of serglycin deficiency on ROS levels would thus be a sign of secretory granule involvement. Indeed, the ROS response was markedly blunted in mast cells lacking serglycin expression (Fig. 1f). Moreover, pre-incubation of mast cells with NAC blocked mefloquine-induced ROS production in WT mast cells (Fig. 1f), whereas only a subtle effect was observed in serglycin^−/−^ mast cells. Hence, these data support that the secretory granules have a central role in the ROS production in response to lysosomotropic agents.

In addition to ROS, reactive nitrogen species such as nitric oxide (NO) can be generated in response to cellular stress^21^. Here we sought to determine whether mefloquine stimulates production of NO in addition to ROS. However, although H_2_O_2_ (positive control) stimulated production of NO in mast cells, mefloquine had a negligible effect (Fig. S1).

### The absence of serglycin causes a delay in mast cell death induced by lysosomotropic challenge

**Fig. 2:**
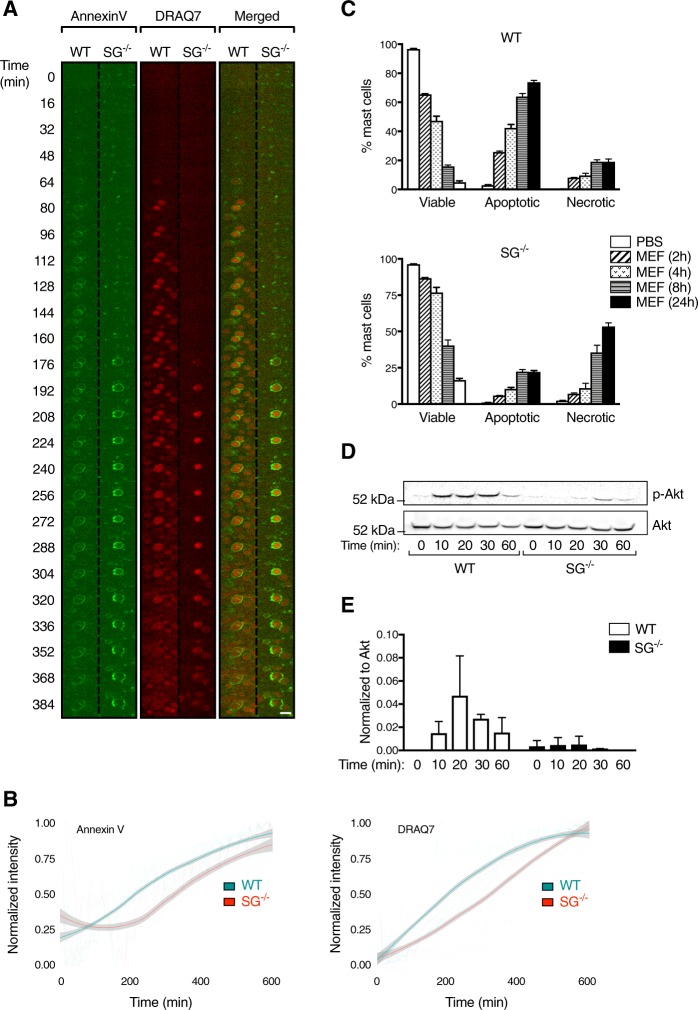
Delayed mefloquine-induced cell death in mast cells lacking serglycin. **a** Representative live confocal images illustrating the expression of cell death markers (Annexin V and DRAQ7) in WT and serglycin^−/−^ (SG^−/−^) mefloquine-treated mast cells. BAM-anchored BMMCs were stained with Annexin V and DRAQ7 and subsequently incubated with mefloquine (20 μM), followed by image recording. Each subframe represents a 62 × 62 µm crop from Supplemental Video 2. Scale bar = 15 µm. **b** Kinetics of Annexin V (left panel) and DRAQ7 (right panel) signals in WT and SG^−/−^ BMMCs. Solid curves show the locally weighted smoothing average for all experiments (WT: *n* = 3, SG^−/−^: *n* = 2) with a 95% confidence interval overlaid. Dotted lines show each technical replicate (WT: *n* = 11, SG^−/−^: *n* = 10). **c** Comparison of cell death in WT and SG^−/−^ mefloquine-treated mast cells. WT and SG^−/−^ mast cells were incubated ± mefloquine (20 μM). At indicated time points, cells were washed, stained with Annexin V and DRAQ7 and analyzed by flow cytometry. Data are shown as means ± SEM. **d** Representative Western blot analysis of Akt and phosphorylated Akt (p-Akt) expression in WT and SG^−/−^ mast cells before and after incubation with mefloquine. **e** Quantitative analysis of p-Akt normalized against total Akt expression. Data are shown as means ± SEM (*n* = 2)

Given that serglycin^−/−^ mast cells were found to produce less ROS in response to mefloquine, we sought to determine whether the absence of serglycin affects the course of mefloquine-induced cell death. To this end, mefloquine-induced cell death was first assessed in WT and serglycin^−/−^ mast cells using live confocal imaging and the cell death markers Annexin V and DRAQ7. Upon incubation with mefloquine, Annexin V and DRAQ7 staining was seen both in WT and serglycin^−/−^ mast cells (Fig. 2a, b; Suppl. Video 2). However, Annexin V/DRAQ7 positivity appeared with a delay in serglycin^−/−^ mast cells compared to their WT counterparts (Fig. 2a, b). As another approach, WT and serglycin^−/−^ mast cells were incubated with mefloquine and cell death was assessed by flow cytometry. Already after 2 h incubation with mefloquine, the proportion of viable cells (Annexin V^−^/DRAQ7^−^) decreased to ∼65% in cultures of WT mast cells (Fig. 2c; upper panel) whereas the proportion of viable cells in cultures of serglycin^−/−^ mast cells was considerably higher (∼87%, *p* < 0.0001; Fig. 2c; lower panel). Similar differences in the ratios of viable/dead cells were also seen after prolonged culture periods (Fig. 2c). These findings thus support that serglycin^−/−^ mast cells are less sensitive to lysosomotropic challenge than WT cells. It was also notable that, whereas WT mast cells predominantly underwent apoptotic cell death (Annexin V^+^/DRAQ7^−^), serglycin^−/−^ cells predominantly underwent necrosis (Annexin V^+^/DRAQ7^+^) (Fig. 2c). These latter findings are in line with previous observations^13^.

The delayed cell death observed in serglycin^−/−^ mast cells prompted us to investigate whether signaling pathways related to cell stress were differentially activated in WT vs. serglycin^−/−^ mast cells. For this purpose, we focused on Akt signaling, which is implicated in cell stress mechanisms^22^. In untreated mast cells, Akt phosphorylation was not detected (Fig. 2d, e). However, prominent Akt phosphorylation was seen in mefloquine-treated WT mast cells, starting ~10 min after mefloquine administration and lasting up to ~60 min (Fig. 2d, e). In contrast, only weak Akt phosphorylation was seen in serglycin^−/−^ mast cells (Fig. 2d, e). These findings suggest that serglycin regulates the intracellular signaling events occurring in response to secretory granule permeabilization.

### Loss of granule acidity prevents mast cell granule damage and cell death in response to lysosomotropic challenge

**Fig. 3:**
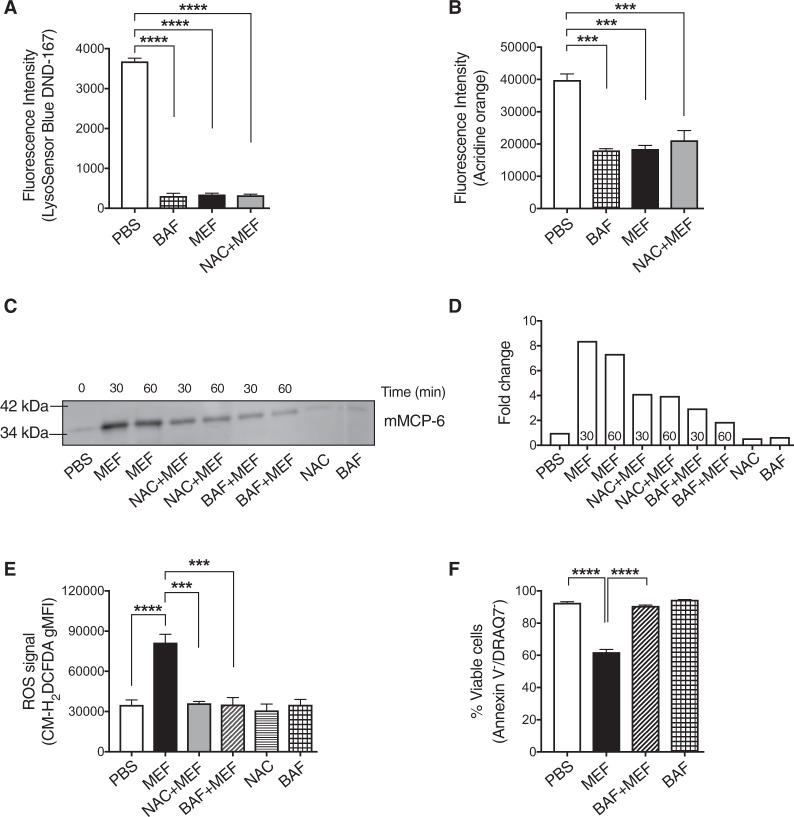
Loss of granule acidity prevents effects of lysosomotropic challenge. **a**–**b** Granule acidification in mefloquine-treated mast cells. Mast cells were incubated ± mefloquine (20 µM) for 30 min or bafilomycin-A1 (BAF; 20 nM) for 3 h. Additionally, NAC-pretreated mast cells were incubated with mefloquine (20 µM) for 30 min. Cells were subsequently incubated with LysoSensor Blue DND-167 (1 μM) for 1 h (**a**) or acridine orange (5 μg/mL) for 15 min (**b**) and granule acidification was assessed using flow cytometry. Data are expressed as means ± SEM. **c** Representative Western blot analysis of mMCP6. Mast cells were preincubated with NAC (8 mM) for 2 h or BAF (20 nM) for 3 h or left untreated, followed by incubation with mefloquine (20 μM). Cells treated with either PBS, NAC or BAF alone were included as controls. At the indicated time points, cytosolic extracts were prepared and subjected to Western blot analysis for presence of the granule protease mMCP6. **d** Quantitative analysis of mMCP6 expression. **e** Effect of BAF on mefloquine-induced ROS generation. Mast cells were preincubated with NAC (8 mM) for 2 h or BAF (20 nM) for 3 h or left untreated, followed by incubation with mefloquine (20 μM). Cells treated with either PBS, NAC or BAF were included as controls. After 30 min, cells were washed and incubated with CM-H_2_DCFDA for 30 min. Subsequently, cells were washed and cellular ROS levels were assessed by flow cytometry. The bars represent means ± SEM of the gMFI for CM-H_2_DCFDA. **f** Effect of BAF on mefloquine-induced cell death in mast cells. Mast cells were preincubated with BAF (20 nM) for 3 h or left untreated, followed by incubation of cells with mefloquine (20 μM). Cells treated with either PBS, NAC or BAF were included as controls. After 2 h, cells were washed and stained with Annexin V and DRAQ7 and cell death was assessed by flow cytometry. Data are expressed as means ± SEM (*n* = 3)

The secretory granules of mast cells contain large amounts of stored proteases, including tryptase, chymase and CPA3^2^. Another hallmark feature of mast cell granules is that they, analogous to lysosomes, are acidic^2^. Based on our finding that ROS production in response to lysosomotropic challenge takes place in the granules, we next assessed the role of ROS in inducing granule membrane damage and whether the acidity of the granules has an impact on this process. To interfere with granule acidification we used bafilomycin-A1, a V-ATPase inhibitor that previously was shown to block granule acidification in mast cells^23^. To monitor granule acidification we used two different probes- LysoSensor and Acridine orange. As seen in Fig. 3a, b, bafilomycin-A1 profoundly impaired granule acidification in mast cells. Moreover, treatment of mast cells with mefloquine caused a dramatic drop in granule acidification (Fig. 3a, b), which was expected considering that granule permeabilization will disrupt the barrier between the acidic granules and the neutral pH milieu of the cytosol^13^. Importantly, preincubation of mast cells with the potent ROS-inhibitor NAC did not prevent the drop in granule acidity in response to mefloquine (Fig. 3a, b), suggesting that the mefloquine-induced de-acidification of the secretory granules is independent of ROS.

To verify that the reduction of LysoSensor and acridine orange signals upon mefloquine treatment is associated with permeabilization of granules, the presence of tryptase was evaluated in cytosolic extracts. Indeed, incubation of mast cells with mefloquine caused a substantial increase in the level of cytosolic tryptase (mMCP6)(Fig. 3c, d). Preincubation of mast cells with NAC had a partial inhibiting effect on the release of granular mMCP6 to the cytosolic compartment (Fig. 3c, d). More strikingly, inhibition of V-ATPase by bafilomycin-A1 caused a profound blockade of the translocation of mMCP6 into the cytosol (Fig. 3c, d). To further explore the role of granule acidity in ROS production and cell death, mast cells were incubated ± mefloquine in the absence or presence of bafilomycin-A1. As expected, treatment of mast cells with mefloquine caused an increase in the generation of ROS and induction of apoptotic cell death (Fig. 3e, f). Intriguingly, pretreatment of mast cells with bafilomycin-A1 totally abolished the mefloquine-induced ROS production and also prevented cell death (Fig. 3e, f). Collectively, these data show that granule acidification is essential for mediating granule permeabilization, ROS production and cell death in response to lysosomotropic challenge.

### Mefloquine-induced ROS production is dependent on iron but independent of NADPH oxidase

**Fig. 4:**
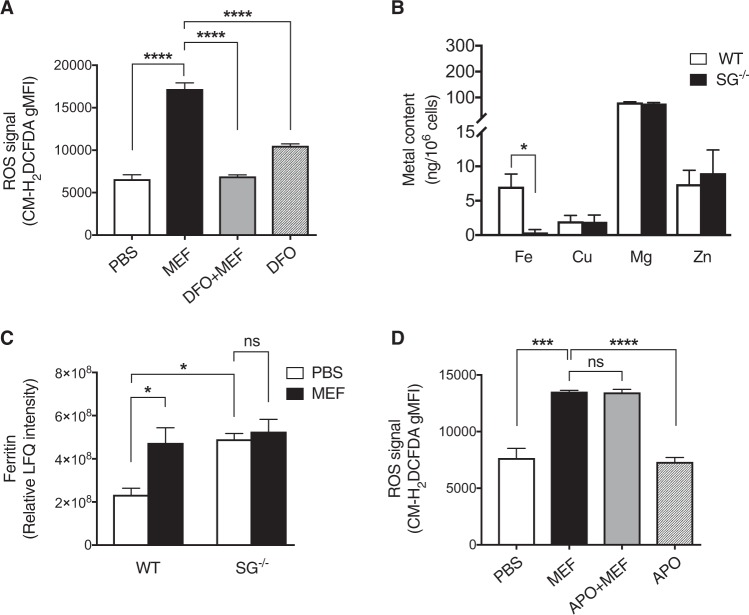
Mefloquine-induced ROS production is dependent on iron. **a** Mast cells were preincubated ± deferoxamine (DFO; 100 μM; for 24 h), followed by incubation ± mefloquine (20 μM). Cellular levels of ROS were assessed by flow cytometry after 30 min. Cells treated with DFO alone were included as controls. The shown data represent means ± SEM (*n* = 3). **b** Total iron, copper, manganese and zinc levels in WT and serglycin^−/−^ (SG^−/−^) mast cells. Metal content was measured by inductively coupled plasma-mass spectrometry. The shown data represent means ± SEM (*n* = 3−5). **c** Relative LFQ intensities for ferritin in PBS- and mefloquine-treated WT and SG^−/−^ mast cells. WT and SG^−/−^ mast cells were incubated ± mefloquine (20 μM) and after 1 h cells were harvested and subjected to MS-based proteomics analysis. The data shown represent means ± SEM (*n* = 4). **d** Mast cells were preincubated ± apocynin (APO; 100 μM) for 24 h, followed by incubation ± mefloquine (20 μM). Cellular levels of ROS were assessed by flow cytometry after 30 min of incubation. Data are presented as means ± SEM (*n* = 3)

Next, we sought to identify the origin(s) of ROS generated in response to granule permeabilization. One candidate intracellular source of ROS is lysosomal iron^24^, from which ROS can be generated through the Fenton reaction. Interestingly, iron-derived ROS production is a contributing factor in mefloquine-induced death of *Plasmodium*^25^, and we therefore considered the possibility that iron could be a source of ROS generated in mefloquine-treated mast cells. To address this possibility we evaluated the impact of the iron chelator deferoxamine mesylate (DFO) on mefloquine-induced ROS production. Indeed, pretreatment of mast cells with DFO blocked ROS generation (Fig. 4a). Based on this notion and on the fact that serglycin^−/−^ mast cells produced less ROS than WT mast cells in response to mefloquine (see Fig. 1f), we next assessed whether serglycin might be involved in the storage of redox active metal ions. To test this possibility, the contents of several metals were measured in WT and serglycin^−/−^ mast cells. There was no difference in the cellular concentrations of Cu, Mg and Zn between WT and serglycin^−/−^ mast cells (Fig. 4b). However, a lower concentration of Fe was observed in serglycin^-/-^ vs. WT mast cells (Fig. 4b). In further support for a role of iron in the cellular responses to lysosomotropic challenge we found by proteomic analysis that ferritin, an iron-binding protein, was markedly upregulated after mefloquine treatment in WT mast cells (Fig. 4c and Suppl. Table 1). This finding is in agreement with previous findings showing that ferritin expression is increased following oxidative stress^26^. In contrast, no significant increase of ferritin was seen in mefloquine-treated serglycin^−/−^ cells (Fig. 4c). Collectively, these data indicate that granular iron, stored in a serglycin-dependent fashion, is involved in ROS production in response to mefloquine.

In addition to redox active metal ions, the NADPH oxidase system is a major pathway for ROS production in many cell types^27^. To determine whether NADPH oxidase contributes to ROS production in the present context we used apocynin, a NADPH oxidase inhibitor. However, no significant effect of NADPH oxidase inhibition on ROS production in mefloquine-challenged mast cells was seen (Fig. 4d).

### A role for MAP kinase signaling in ROS production downstream of secretory granule permeabilization

**Fig. 5:**
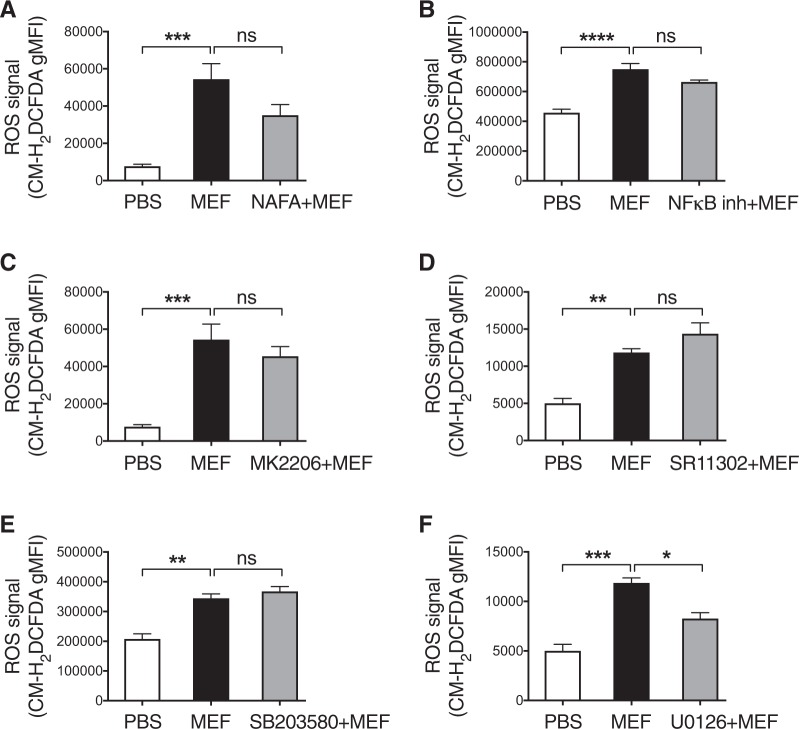
The ERK1/2 pathway contributes to ROS generation in response to secretory granule permeabilization. **a**-**f** Effect of tryptase and cell signaling pathways on mefloquine-induced ROS generation in mast cells. Mast cells were preincubated ± either nafamostat mesylate (NAFA; 1 μM), NF-κB Activation Inhibitor (NF-κB inh; 0.1 μM), MK2206 (1 μM), SR11302 (10 μM), SB203580 (10 μM), or U0126 (10 μM) for 2 h, followed by incubation of cells ± mefloquine (20 μM). Cellular levels of ROS were assessed by flow cytometry after 30 min. The data shown represent means ± SEM (*n* = 3−4)

In an attempt to further explore the downstream signaling events involved in mefloquine-induced oxidative stress, ROS production in mast cells treated with a panel of inhibitors of candidate pathways was evaluated (Fig. 5). No significant reduction of ROS production was seen after incubation with inhibitors of either tryptase (Nafamostat), NFκB, Akt (MK2206), AP-1 (SR11302) or P38 MAP kinase (SB203580)(Fig. 5a–e). However, a significant inhibitory effect was seen using an inhibitor of the ERK1/2 MAPK kinase pathway (U0126; inhibitor of MEK1/2)(Fig. 5f). Hence, these data indicate that the ERK1/2 MAP kinase signaling pathway contributes to ROS production. Neither of the used inhibitors, administered in the absence of mefloquine, had any significant impact on ROS production (Suppl. Fig. 2).

### Granzyme B has a key role in ROS production in response to lysosomotropic challenge

**Fig. 6:**
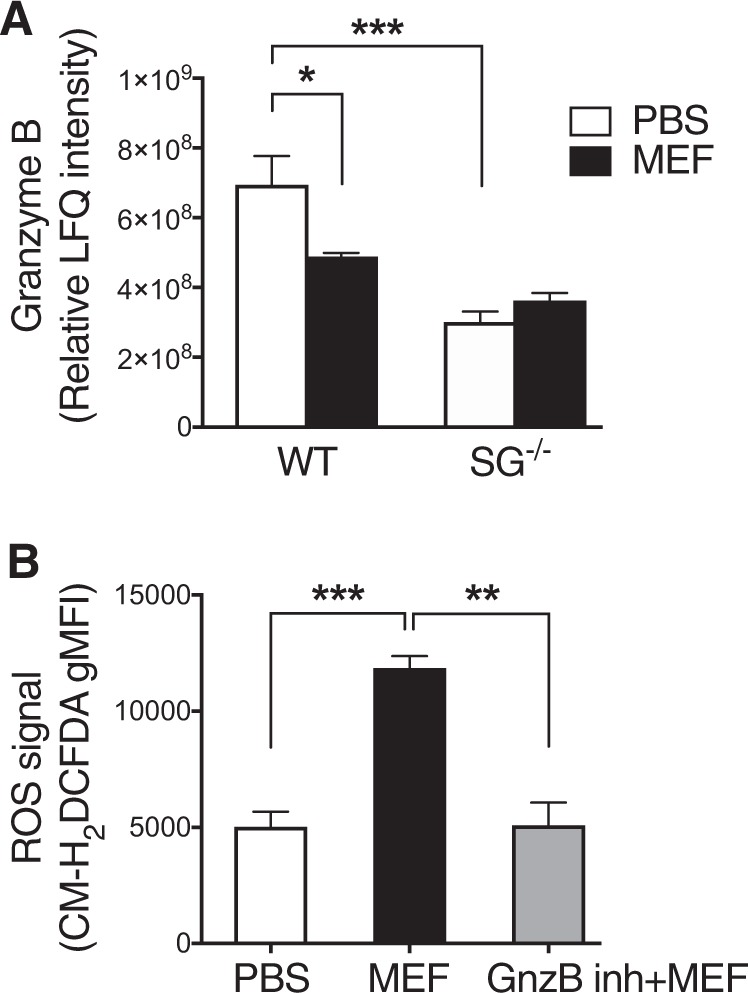
Granzyme B contributes to ROS generation in response to secretory granule permeabilization. **a** Relative LFQ intensities for granzyme B in PBS and mefloquine-treated WT and serglycin^−/−^ (SG^−/−^) mast cells. WT and SG^−/−^ mast cells were incubated ± mefloquine (20 μM) and after 1 h cells were harvested and subjected to proteomics analysis. The data shown represent means ± SEM (*n* = 4). **b** Effect of granzyme B on mefloquine-induced ROS generation in mast cells. Mast cells were preincubated ± granzyme B inhibitor IV (GnzB inh; 10 μM) for 2 h, followed by incubation of cells ± mefloquine (20 μM). ROS levels were assessed by flow cytometry after 30 min. The data shown represent means ± SEM (*n* = 3)

To gain more detailed insight into the molecular events occurring after lysosomotropic challenge of mast cells, we employed proteomic analysis of WT vs. serglycin^−/−^ mast cells, before and after treatment with mefloquine. As seen in Suppl. Table 1, a large number of proteins were downregulated at least 2-fold in WT mast cells following mefloquine treatment, whereas no such effects were seen in serglycin^−/−^ cells. One of the proteins that were differentially affected in WT vs. serglycin^−/−^ cells was granzyme B, being significantly downregulated in WT cells subjected to mefloquine, but not affected in serglycin^−/−^ cells (Fig. 6a). We also noted that the levels of granzyme B in naïve cells were significantly higher in WT vs. serglycin^−/−^ mast cells (Fig. 6a). Since granzyme B has a known pro-apoptotic function^28^ we considered the possibility that granzyme B could have an impact on ROS production in response to lysosomotropic challenge. To assess this possibility, we measured ROS production in mast cells treated with a specific granzyme B inhibitor. The results revealed a completely abrogated ROS production under these conditions (Fig. 6b). Taken together, these data indicate that granzyme B has a key role in the ROS production following lysosomotropic challenge of mast cells.

## Discussion

Mast cells are notorious for their detrimental impact in allergic settings, but also in a variety of additional disorders^29,30^. Strategies to limit harmful mast cell actions are therefore needed. To meet this demand we have recently established a principle for inducing selective apoptosis in mast cells through the use of lysosomotropic agents^13–16,31^. Lysosomotropic agents are known to enter lysosomes and then cause membrane permeabilization leading to efflux of lysosomal compounds into the cytosol followed by triggering of cell death^32^. Since mast cell granules have similar properties as lysosomes, i.e., being acidic and containing various lysosomal hydrolases^2^, we reasoned that mast cell granules might be sensitive to this class of compounds. Indeed, we have shown that lysosomotropic agents potently induce mast cell secretory granule permeabilization leading to apoptotic cell death^13–16,31^. Intriguingly, mast cells are more sensitive to this type of cell death than are a panel of other cells types^13–16,31^, and lysosomotropic agents have therefore emerged as selective inducers of mast cell apoptosis with therapeutic implications.

In previous studies we have shown that apoptotic cell death in response to lysosomotropic challenge is associated with robust production of ROS^13,16^. However, the mechanism of ROS production in this setting had not been revealed. Here we show that the ROS production in response to lysosomotropic challenge predominantly takes place within the secretory granules. As a major evidence for this notion, live imaging analysis revealed that mefloquine-induced ROS production shows a strong co-localization with secretory granule markers. In addition, ROS production and cell death in response to mefloquine was markedly attenuated in mast cells lacking serglycin, a secretory granule-restricted proteoglycan^20^. Finally, ROS production in response to mefloquine could be completely abolished by interference with granule acidification.

Another major question was to identify the actual source(s) of ROS produced after challenge with lysosomotropic agent. As one of the candidates we considered the NADPH oxidase system, which is known as a major pathway for ROS production, in particular in phagocytic cells. Redox active metals, e.g., iron, are other candidate sources for ROS production. Such metals can give rise to ROS by undergoing the Fenton reactions in which electrons are non-enzymatically donated to oxygen. Of these candidates, the NADPH oxidase system appeared to be of minor importance. By contrast, our findings revealed a significant reduction of ROS production in the presence of DFO, an iron chelator, suggesting that iron is a significant source for ROS production in response to granule permeabilization. Importantly, DFO does not diffuse over the cell membrane into the cytosol but is rather taken up by fluid phase endocytosis and then enters lysosomal compartments^33^. Hence, DFO taken up by mast cells will most likely enter the secretory granules, and the blunted ROS response in the presence of DFO will thus represent effects on granular iron depots (rather than on cytosolic iron).

The exact mechanism(s) behind the effect of serglycin-deficiency on ROS production is intriguing. Interestingly, our findings suggest that the absence of serglycin causes a reduction in the amount of iron stored in mast cells. Serglycin is highly negatively charged due to a high density of sulfate and carboxyl groups on the glycosaminoglycan chains attached to the serglycin protein core^34^. It thus appears plausible that serglycin can engage in electrostatic interactions with cationic iron. The reduction in ROS production in serglycin^−/−^ mast cells could consequently reflect an indirect effect of serglycin on iron storage. Serglycin is also known to promote the storage of a variety of other compounds through electrostatic interactions, including various proteases such as tryptase, chymase, CPA3 and also granzyme B. Granzyme B is a major pro-apoptotic protease present in cytolytic granules of cytotoxic T lymphocytes^28^, but is also expressed at high levels in mast cells^35^. Interestingly, we found that granzyme B was stored at lower levels in serglycin^−/−^ vs. WT mast cells and was significantly depleted following mefloquine addition to WT but not serglycin^−/−^ mast cells. Moreover, ROS production in response to lysosomotropic challenge was markedly reduced after inhibition of granzyme B activity. Collectively, these findings suggest that granzyme B has a role in ROS reduction following lysosomotropic challenge and that the reduced ROS production in serglycin^−/−^ cells can be partially explained by reduced granzyme B storage.

A striking finding in this study was that interference with granule acidification, by inhibition V-ATPase, caused an essentially complete inhibition of ROS production in response to lysosomotropic challenge. Moreover, V-ATPase inhibition effectively inhibited granule permeabilization and also prevented cell death. Hence, granule acidification has a major impact on all of the downstream events occurring after lysosomotropic challenge of mast cells. We cannot with certainty explain the detailed chain of events behind these findings. However, a plausible scenario is that low pH in the mast cell secretory granules promotes membranolytic effects of mefloquine. For example, we may envisage that acidic pH promotes molecular rearrangements in mefloquine in granules, giving rise to membrane-perturbing properties. This would be in analogy with the presumed action of other lysosomotropic agents, which after protonation in acidic compartments gain surfactant-like properties^36^.

In summary, this study outlines the mechanism of ROS production in mast cells subjected to lysosomotropic challenge, and identifies a role for granule acidification in the pro-apoptotic events occurring in mast cells following secretory granule permeabilization.

## Materials and Methods

### Cell culture

Bone marrow-derived mast cells (BMMCs) were generated as previously described^37^. Briefly, bone marrow cells were isolated from wild type (WT) and/or serglycin-deficient (serglycin^−/−^) mice on C57BL/6 genetic background and grown at 37 °C with 5% CO_2_ in Dulbecco’s modified Eagle’s medium (Sigma-Aldrich) containing 30% WEHI-3B-conditioned medium, 10% heat-inactivated fetal bovine serum (Gibco, Carlsbad, CA), 100U/ml penicillin, 100 μg/ml streptomycin, 2 mM L-glutamine (all from Sigma-Aldrich) and 10 ng/ml IL-3 (PeproTech, Rocky Hill, NJ). The medium was changed twice every week and cells were cultured at a concentration 0.5 × 10^6^ cells/ml for at least 4 weeks to obtain mature and pure BMMCs. The animal experiments were approved by the local ethical committee (Uppsala djurförsöksetiska nämnd; Dnr 5.8.18-05357/2018).

### Flow cytometry assessment of cell death

Cells were washed and resuspended in Annexin V binding buffer (BD Biosciences, Franklin Lakes, NJ) and stained with Annexin V (BD Biosciences) and DRAQ7™ (Biostatus Ltd., Shepshed, UK). Subsequently, stained cells were analyzed with an LSRFortessa or Accuri flow cytometer (BD Biosciences) for assessment of cell death. Data analysis was performed using the FlowJo software (TreeStar Inc., Ashland, OR).

### Measurement of reactive oxygen species (ROS) and nitric oxide (NO)

For ROS measurement, mast cells (0.5 × 10^6^ BMMCs/well) were preincubated in the absence or presence of various inhibitors or iron chelator. Subsequently, cells were incubated with either mefloquine or PBS, or left untreated for 30 min. Cells were then washed and incubated with 5 μM CM-H_2_DCFDA for 30 min at 37 °C in dark. After a brief wash to remove excess extracellular probe, the cellular ROS levels were assessed by flow cytometry. For NO measurement, mast cells were incubated with mefloquine, PBS or hydrogen peroxide (as positive control) for indicated time points. After a brief wash, cells were incubated with 5 μM DAF-FM Diacetate for 20 min at 37 °C. Cells were washed and incubated in fresh media for 30 min at 37 °C to allow complete de-esterification of the intracellular diacetates before measuring fluorescence intensity by flow cytometry.

### Measurement of GSH/GSSG ratio

The ratio of reduced to oxidized glutathione (GSH/GSSG ratio) was assessed using a GSH/GSSG-Glo™ Assay (Promega, Madison, WI) according to the manufacturer’s instructions. Briefly, mast cells (0.5 × 10^6^ BMMCs/well) were incubated with PBS or mefloquine for 30 min followed by incubation with Total Glutathione Lysis Reagent. After 5 min of shaking, cell lysates were incubated with Luciferin Generation Reagent (for 30 min) and thereafter with Luciferin Detection Reagent (for 15 min). Subsequently, luminescence signals were measured using a TECAN microplate reader and GSH/GSSG ratio was determined.

### Measurement of granule pH

Untreated or NAC-pretreated mast cells (0.5 × 10^6^ BMMCs/well) were incubated with either PBS (for 30 min), bafilomycin-A1 (for 3 h), or mefloquine (for 30 min). Subsequently, LysoSensor Blue DND-167 (1 μM) was added to the cells and incubated for 1 h at 37 °C. Cells were washed and resuspended in PBS and fluorescence was measured by flow cytometry to assess granule pH. Alternatively, granule pH was measured using acridine orange. For this, cells were treated as described above and subsequently incubated with 5 μL acridine orange for 15 min at 37 °C. Cells were extensively washed and resuspended in 500 μL of PBS. 100 μL aliquots of the cell suspension were transferred to a 96-well plate and fluorescence was measured with excitation at 485 nm and emission at 650 nm, using a TECAN microplate reader.

### Western blot analysis

Mast cells (2 × 10^6^ BMMCs) were harvested after the indicated treatments and were lysed with a lysis buffer (1% Triton X-100, 0.1% SDS, and 1 mM EDTA in PBS (pH 7.4)) in the presence of Pierce phosphatase inhibitor (Thermo Fisher Scientific, Waltham, MA) and protease inhibitor cocktail mixture (Roche Diagnostics, Mannheim, Germany) for 30 min on ice. The cell debris was removed by centrifugation (14,000 rpm, 20 min, 4 °C) and the supernatant was collected for Western blot analysis of Akt and phosphorylated Akt (p-Akt) using anti-Akt and anti-p-Akt antibodies (Phospho-Akt (Ser473) antibody; Cat#9271; Cell Signaling Technology, Danvers, MA). Alternatively, cytosolic extracts were prepared for Western blot analysis of mMCP-6. For this, harvested mast cells were resuspended in ice-cold digitonin extraction buffer (10 μg/ml digitonin, 250 mM sucrose, 20 mM HEPES, pH 7.5, 10 mM KCl, 1.5 mM MgCl_2_, 1 mM EDTA, 1 mM EGTA) for 10 min on ice. The cell debris was removed by centrifugation (13,000 rpm, 2 min, 4 °C) and the supernatant was collected for Western blot analysis of mMCP-6 using anti-mMCP-6 antisera (raised in rabbits) and fluorescently labeled secondary antibodies. The membranes were scanned using an Odyssey CLX imaging system (LI-COR Biosciences, Lincoln, NE) and protein signals quantified using Image Studio Software (LI-COR) according to the manufacturer’s instructions.

### Confocal microscopy

To monitor and localize ROS production by live confocal imaging, mast cells (1.5 × 10^6^ cells) were immobilized overnight in MatTek glass bottom microwell dishes (MatTek Co., Ashland, MA) using a biocompatible anchor for membrane (BAM; SUNBRIGHT® OE-040CS, NOF Corporation, Tokyo, Japan). To label granules and detect ROS, cells were incubated with a cocktail of probes (50 nM LysoTracker^TM^ Red DND-99 and 5 μM CellROX^TM^ Deep Red) for 30 min. Cells were subsequently washed and kept in PBS. Images were immediately recorded at the indicated time intervals at 37 °C and 5% CO_2_ at baseline (initial 72 min), and after addition of mefloquine (for another 112 min) using a Nikon Ti2-E microscope, equipped with an X-LIGHT V2 L-FOV spinning disk with a pinhole size of 60 µm (Crest Optics). A 100X/1.45 NA oil objective (Nikon) and a Prime 95B 25 mm camera (Photometrics) were used to capture the images. To monitor cell death by live confocal imaging, BAM-anchored WT and serglycin^−/−^ mast cells (1.5 × 10^6^ cells) were incubated with a cocktail of Annexin V and DRAQ7 in a glass bottom 6-well plate (MatTek Co.). Thereafter, cells were treated with either PBS or mefloquine and images were immediately recorded at indicated time intervals, at 37 °C and 5% CO_2_, through a 40X/0.6 NA air objective on a Nikon Ti2-E microscope.

### Image analysis

#### Colocalization analysis

The ImageJ plugin Coloc 2 (v. 3.0.0) was used to calculate the Manders coefficients, M1 and M2, between the LysoTracker and CellROX channel images. Images were automatically thresholded using the Costes method.

#### Annexin V and DRAQ7 intensity kinetics

The time lapse movies were processed with a custom analysis script in ImageJ that did the following:

1. A cell mask was created by applying a 4-pixel variance filter to the DIC channel, followed by default Huang thresholding and a binary Close-operation.
2. Average masked intensities were recorded in both fluorescence channels for each time point.

The csv files generated by the analysis macro were further processed in R (v. 3.5.1), where the intensities in each channel were normalized to the range 0–1, and plots were generated using ggplot2 (v. 3.1.0). Analysis macro code is available from the authors on request.

### Metal measurements

Metal content was measured using inductively coupled plasma–mass spectrometry (NexION 300D, PerkinElmer) as previously described^38^. Briefly, mast cells (1 × 10^6^ cells) were washed with PBS and cell pellets were digested in HNO_3_ (Sigma-Aldrich) overnight. Subsequently, the mixtures were boiled at 85 °C for 30 min, cooled down at room temperature for 1 h, and after addition of H_2_O, metal content was analyzed by mass spectrometry.

### Proteomics analysis

#### Sample preparation

WT and serglycin^−/−^ mast cells (6 × 10^6^ BMMCs) were harvested 1 h after treatment with PBS or mefloquine and were lysed in a 1% β-octyl glucopyranoside and 6 M urea-containing buffer according to a standard operating procedure. The total protein content in the samples was measured using the DC Protein Assay Kit (BioRad Laboratories, Hercules, CA) with bovine serum albumin (BSA) as standard. Aliquots corresponding to 25 μg protein were taken out for digestion. The proteins were reduced, alkylated, in-solution digested by trypsin and desalted by a spin filter aided standard operating procedure as previously described^39^. The collected peptide filtrates were vacuum centrifuged to dryness using a Speedvac system ISS110 (Thermo Scientific, Waltham, MA). The samples were dissolved in 40 μL 0.1% formic acid (FA) and further diluted four times prior to LC-MS/MS analysis.

#### LC-MS/MS

The resulting peptides were separated in reversed-phase on a C18-column, applying a 150 min long gradient, and electrosprayed on-line to a QEx-Orbitrap mass spectrometer (Thermo Finnigan). Tandem mass spectrometry was performed applying HCD.

#### Qualitative analysis

Database searches were performed using MaxQuant (version 1.5.1.2). The search was set towards proteins from the *Mus musculus* proteome extracted from UniProt (release November 2017). The search parameters were set to Taxonomy: *Mus musculus*, Enzyme: Trypsin. Fixed modification was Carbamidomethyl (C), and variable modifications were Oxidation (M), Deamidation (NQ). The search criteria for protein identification were set to at least two matching peptides.

#### Quantitative analysis

Acquired RAW-data files were quantitatively analyzed by MaxQuant (version 1.5.1.2). Protein identification was performed by a search against the same database as for the qualitative analysis. The results of all samples were combined to total label-free protein quantification (LFQ) intensities for each sample. Proteins with *p* value lower than 0.05, showing fold change of at least 2 (cut off ratio for up-regulated of 2 and down-regulated of 0.5) are shown in Suppl. Table 1.

### Statistical analysis

Statistical differences between groups were determined using one-way ANOVA with post hoc Tukey’s multiple comparison test unless otherwise stated. *p*-values of less than 0.05 were considered significant (*p < 0.05; **p < 0.01; ***p < 0.001; ***p < 0.0001). The graphs were prepared and statistics calculated using GraphPad Prism 7.0 (GraphPad software Inc., San Diego, CA).

## Supplementary information

Suppl Fig 1

Suppl Fig 2

Suppl Table 1

Suppl Video 1

Suppl Video 2

Supplemental Material File #1
