# Supplementary figures and images for: Lysosomotropic challenge of mast cells causes intra-granular reactive oxygen species production
Source: Cell Death Discov. 2019 May 15;5:95. doi: 10.1038/s41420-019-0177-3 (PMC6520368; doi:10.1038/s41420-019-0177-3)

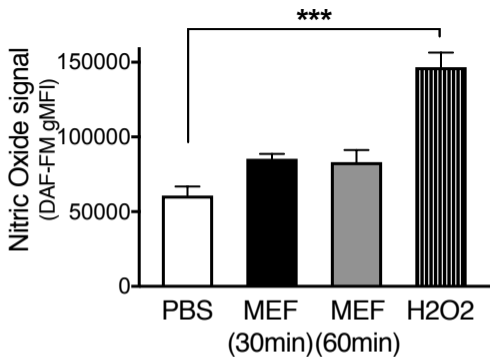

**Figure S1**

Supplement: Supplementary file 1 — Suppl Fig 1 [file 41420_2019_177_MOESM1_ESM.pdf]

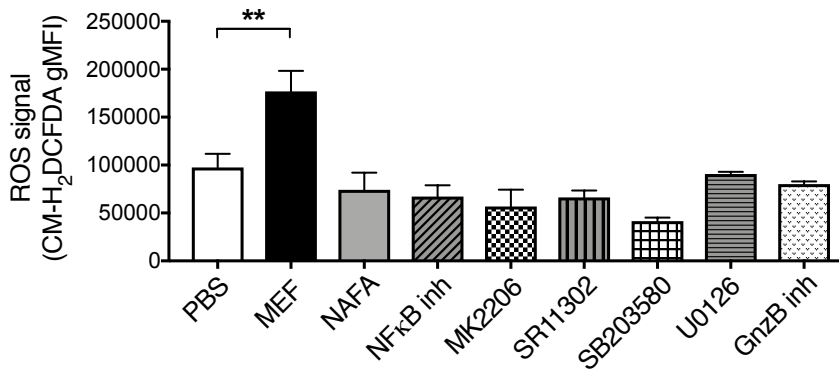

**Figure S2**

Supplement: Supplementary file 2 — Suppl Fig 2 [file 41420_2019_177_MOESM2_ESM.pdf]
