# Supplementary material for: Lysosomotropic challenge of mast cells causes intra-granular reactive oxygen species production
Source: Cell Death Discov. 2019 May 15;5:95. doi: 10.1038/s41420-019-0177-3 (PMC6520368; doi:10.1038/s41420-019-0177-3)
Supplement: Supplementary file 6 — Supplemental Material File #1 [file 41420_2019_177_MOESM6_ESM.docx]

**Supplementary Material**

**Suppl. Figure S1. Mefloquine does not induce nitric oxide (NO) production in mast cells.**

Mast cells were incubated either with PBS, mefloquine (MEF; 20 μM) or hydrogen peroxide (H_2_O_2_; 1 mM). After 30 or 60 min, cells were washed and incubated with DAF-FM diacetate (5 μM) for 15 min. Subsequently, cells were briefly washed to remove the excess extracellular probe, and the cellular NO levels were immediately assessed by flow cytometry. The bars represent means ± SEM of the geometric mean fluorescence intensity (gMFI) for DAF-FM (n = 2-3).

**Suppl. Figure S2. Inhibitors alone do not induce ROS generation at the concentration used.** Mast cells were incubated with either PBS, mefloquine (MEF; 20 μM), nafamostat mesylate (NAFA; 1 μM), MK2206 (1 μM), U0126 (10 μM), SB203580 (10 μM), SR11302 (10 μM), NF-κB Activation Inhibitor (NF-κB inh; 0.1 μM) or granzyme B Inhibitor IV (GnzB inh; 10 μM). Cellular levels of ROS were assessed by flow cytometry after 30 min. Data are presented as means ± SEM (n = 3).

**Suppl. Table 1. Proteome analysis of mast cells.** WT and serglycin^-/-^ mast cells were incubated ± mefloquine for 1h. Cells were recovered and analyzed by LC-MS/MS-based proteomics (see Materials and Methods). Proteins that were significantly up- or down-regulated at least 2-fold after mefloquine treatment are displayed. Comparisons of protein levels were made between the different groups (WT vs. serglycin^-/-^; non-treated vs. treated). As displayed, significant effects (≥ 2-fold) on protein levels were seen when comparing either non-treated WT vs. serglycin^-/-^ mast cells, non-treated vs. treated WT mast cells and between treated WT vs. serglycin^-/-^ mast cells. No significant, more than 2-fold, effects on protein levels were detected when comparing non-treated vs. treated serglycin^-/-^ mast cells.

**Suppl. Video 1.**

Representative live confocal time lapse movie illustrating the granule damage and production of ROS in mefloquine-treated mast cells. BAM-anchored bone marrow-derived mast cells (BMMCs) were preincubated with LysoTracker Red DND-99 (red) and CellROX Deep Red (green) for 30 min at 37°C. Cells were then washed and live confocal imaging was performed immediately. Image acquisition starts 72 min prior to the addition of mefloquine. At 00:00 20 μM of mefloquine was added to the cells and image recording continued for 112 min. The movie represents a 12x12 µm area and the white digits indicate time before/after the addition of mefloquine (hh:mm).

**Suppl. Video 2.**

Representative live confocal time lapse movie illustrating the expression of cell death markers Annexin V (green) and DRAQ7 (red) in WT (top) and serglycin^-/-^ (SG^-/-^, bottom) mefloquine-treated mast cells. White digits indicate time after the addition of mefloquine (hh:mm).
